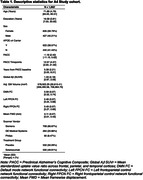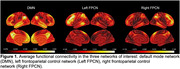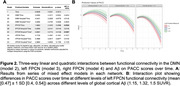# Left frontoparietal control network connectivity moderates the effect of amyloid on cognitive decline in preclinical Alzheimer’s disease

**DOI:** 10.1002/alz.093913

**Published:** 2025-01-09

**Authors:** Rory Boyle, Zahra Shirzadi, Gillian T Coughlan, Mabel Seto, Michael J Properzi, Hannah M Klinger, Diana L Townsend, Ziwen Yuan, Catherine E Scanlon, Roos J Jutten, Kathryn V Papp, Rebecca E Amariglio, Keith A Johnson, Dorene M Rentz, Jasmeer P. Chhatwal, Reisa A Sperling, Aaron P Schultz, Rachel F Buckley

**Affiliations:** ^1^ Massachusetts General Hospital, Harvard Medical School, Boston, MA USA; ^2^ Center for Alzheimer Research and Treatment, Brigham and Women’s Hospital, Harvard Medical School, Boston, MA USA; ^3^ Brigham and Women’s Hospital, Boston, MA USA

## Abstract

**Background:**

Stronger default mode (DMN) and bilateral frontoparietal control network (FPCN) resting‐state functional connectivity are associated with reduced ß‐amyloid (Aß)‐related cognitive decline in cognitively unimpaired older adults, who were predominantly Aß negative. This suggests that these networks might support cognitive resilience in the face of early AD pathology but it remains unclear whether these effects are apparent in preclinical AD. We investigated whether left‐FPCN, right‐FPCN, and DMN connectivity moderated the effect of Aß on cognitive decline using a large multi‐site dataset from the Anti‐Amyloid Treatment in Asymptomatic Alzheimer’s Disease (A4) study.

**Method:**

Cognitively unimpaired participants with elevated Aß on screening amyloid‐PET (n = 1,062, mean age = 71.9 years, 60% women, see Table1), underwent functional MRI (3T) at baseline. We obtained connectivity estimates of the left‐FPCN, right‐FPCN, and DMN using template‐based rotation (Fig.1). Cognition was measured using the Preclinical Alzheimer’s Cognitive Composite (PACC; average follow‐up including open‐label extension period = 280 weeks [SD = 115]). We examined three‐way linear and quadratic interactions between functional connectivity and Aß burden with time in mixed effects models (model 2 = DMN, 3 = left‐FPCN, 4 = right‐FPCN). 1. PACC∼time + covariates×time 2‐4A. PACC∼FC×time + covariates×time 2‐4B. PACC∼FC×Aß×time + covariates×time 2‐4C. PACC∼FC×Aß×time + FC×Aß×time2 + covariates×time Covariates included age, education, APOEe4 status, ICV‐adjusted gray matter volume, treatment group (Solanezumab vs placebo), and head motion. We modeled random effects of intercept and slope for each participant and included scanner as a random effect.

**Result:**

The three‐way quadratic interaction of connectivity and Aß burden with time was statistically significant for the left FPCN (p‐value corrected for multiple comparisons = .047, Fig.2), with a moderate effect size. That is, stronger left FPCN connectivity was associated with less cognitive decline in those with elevated Aß. The pattern of effects were similar, but not statistically significant once all covariates were included, for the right FPCN and DMN.

**Conclusion:**

In a very large multi‐site clinical trial dataset, individuals with stronger left FPCN connectivity showed reduced Aß‐related cognitive decline. This effect was specific to the left‐FPCN, in line with previous cross‐sectional findings. Our finding demonstrates that this protective effect of left FPCN connectivity can be observed in preclinical AD.